# Chronic multisite pain in major depression and bipolar disorder: cross-sectional study of 149,611 participants in UK Biobank

**DOI:** 10.1186/s12888-014-0350-4

**Published:** 2014-12-10

**Authors:** Barbara I Nicholl, Daniel Mackay, Breda Cullen, Daniel J Martin, Zia Ul-Haq, Frances S Mair, Jonathan Evans, Andrew M McIntosh, John Gallagher, Beverly Roberts, Ian J Deary, Jill P Pell, Daniel J Smith

**Affiliations:** Institute of Health & Wellbeing, University of Glasgow, Glasgow, Scotland UK; Division of Psychiatry, University of Edinburgh, Edinburgh, UK; Centre for Cognitive Ageing and Cognitive Epidemiology, Department of Psychology, University of Edinburgh, Edinburgh, UK

**Keywords:** Major depression, Bipolar disorder, Chronic pain, Comorbidity

## Abstract

**Background:**

Chronic pain has a strong association with major depressive disorder (MDD), but there is a relative paucity of studies on the association between chronic multisite pain and bipolar disorder (BD). Such studies are required to help elucidate the complex biological and psychological overlap between pain and mood disorders. The aim of this study is to investigate the relationship between chronic multisite pain and mood disorder across the unipolar-bipolar spectrum.

**Methods:**

We conducted a cross-sectional study of 149,611 UK Biobank participants. Self-reported depressive and bipolar features were used to categorise participants into MDD and BD groups and a non-mood disordered comparison group. Multinomial logistic regression was used to establish whether there was an association between extent of chronic pain (independent variable) and mood disorder category (dependent variable), using no pain as the referent category, and adjusting for a wide range of potential sociodemographic, lifestyle and comorbidity confounders.

**Results:**

Multisite pain was significantly more prevalent in participants with BD and MDD, for example, 4–7 pain sites: BD 5.8%, MDD 4.5%, and comparison group 1.8% (p < 0.001). A relationship was observed between extent of chronic pain and risk of BD and persisted after adjusting for confounders (relative to individuals with no chronic pain): 2–3 sites RRR of BD 1.84 (95% CI 1.61, 2.11); 4–7 sites RRR of BD 2.39 (95% CI 1.88, 3.03) and widespread pain RRR of BD 2.37 (95% CI 1.73, 3.23). A similar relationship was observed between chronic pain and MDD: 2–3 sites RRR of MDD 1.59 (95% CI 1.54, 1.65); 4–7 sites RRR of MDD 2.13 (95% CI 1.98, 2.30); widespread pain RRR of MDD 1.86 (95% CI 1.66, 2.08).

**Conclusions:**

Individuals who report chronic pain and multiple sites of pain are more likely to have MDD and are at higher risk of BD. These findings highlight an important aspect of comorbidity in MDD and BD and may have implications for understanding the shared neurobiology of chronic pain and mood disorders.

**Electronic supplementary material:**

The online version of this article (doi:10.1186/s12888-014-0350-4) contains supplementary material, which is available to authorized users.

## Background

Chronic pain is known to be associated with depressive features and major depressive disorder (MDD) [1,2] but the association between chronic pain and bipolar disorder (BD) has received less attention. Very few studies have investigated associations between chronic *multisite* pain (pain occurring at two or more body sites [3]) and mood disorders, although there is some evidence of a positive correlation between number of chronic pain sites and depressive features [4].

A study of the health records of 5 million US Veterans Affairs patients found that those with MDD or BD were 1.8 and 2.6 times more likely, respectively, to have a diagnosis of one or more painful conditions in their health records than non-mood disordered controls [5]. The prevalence of chronic pain was reported to be 23.7% in a meta-analysis of chronic pain in BD studies; and patients with BD were found to be at a two-fold increased risk of having chronic pain compared to the general population [6]. Two further studies investigating the association between chronic pain and BD suggested that pain may be under-recognised among individuals with BD. Cerimele and colleagues have reported that in a cohort of primary care patients receiving an intervention for BD 46% were also receiving treatment for pain or experiencing pain that interfered with their daily functioning [7]. In the Singapore Mental Health Study, chronic pain was the most prevalent comorbid physical condition among participants with BD, affecting 40.4% of these individuals [8]. These initial findings support the need for a better understanding of the relationships between chronic pain and mood disorder in order to improve the clinical management of these challenging to treat conditions. Further epidemiological understanding will lead to the development of biological hypotheses that might explain why chronic pain and mood disorder (depression and/or bipolar disorder) commonly co-occur.

UK Biobank is a large, prospective cohort study of over 500,000 participants recruited from the general population in England, Scotland and Wales [9]. It represents a unique opportunity to investigate associations (and, in future, health outcomes such as onset and prognosis of disease, and shared neurobiological and genetic components) between chronic pain syndromes and mood disorders. The extent of baseline data collected also makes it possible to take account of a wide range of potential confounders including sociodemographic, lifestyle, psychological and medical comorbidity factors.

In this cross-sectional study, we use baseline UK Biobank data to compare the nature and extent of chronic pain among participants with features of MDD and BD compared to those without significant mood disorder features. We hypothesized that multisite and widespread chronic pain would be common in MDD and possibly more common in BD, relative to the comparison group, and that these relationships would be independent of potential confounding factors.

## Methods

### Study design

UK Biobank is a large, population-based cohort of approximately 502,600 UK residents aged 40–69 years at recruitment. It is one of the world’s largest Biobank cohorts, designed to investigate the aetiology, prognosis, detection and management of a large number of chronic conditions over time [9]. At baseline assessment, all participants provided detailed self-reported information on demographics, health and lifestyle factors, alongside physical measurements, and blood, saliva and urine samples, which will be analysed in future studies. The current study uses baseline data on 172,745 participants who completed detailed questions on lifetime depressive and manic features. These mood disorder assessments were added during the final two years of UK Biobank recruitment (2008–2010), representing 34.4% of the cohort.

### Participants and data collection

All adults registered with the UK National Health Service, who were aged 40–69 years and lived within 25 miles of one of 22 assessment centres across the UK, were invited to participate in UK Biobank [10]. Individuals who agreed to participate attended the assessment centre where they provided full informed consent. Participants then completed a detailed touch-screen questionnaire which gathered demographic, lifestyle and medical information and undertook a nurse-led interview which gathered more detailed information on medical conditions and medications. Details of the data used in this study are provided below.

### Classification of mood disorder

We have previously reported on the classification and internal validity of probable MDD and BD within UK Biobank, using the self-reported data collected via the baseline touch-screen questionnaire [11]. These diagnostic classifications are summarised below in the “Detailed information of the UK Biobank variables used for classifying lifetime experience of probable bipolar disorder and depression” subsection. In brief, BD included participants who met the criteria for BD type I or type II; MDD included participants who met the criteria for recurrent severe, recurrent moderate or single episode depression; and all other participants who provided complete data comprised the non-mood disordered comparison group, that is they were free of probable MDD and BD according to our criteria. Mood groups were mutually exclusive; participants who met criteria for both BD and MDD were included in the BD group. For ease of reference the term “mood disorder groups” is used throughout the manuscript to refer to the three groups of interest – non-mood disordered comparison group, probable MDD and probable BD. These classifications were based on a lifetime history of manic and depressive symptoms.

#### Detailed information of the UK Biobank variables used for classifying lifetime experience of probable BD and MDD

Criteria for a classification of lifetime experience of probable BD:

1. **BD, type I:** Ever ‘manic or hyper’ for 2 days OR ever ‘irritable/argumentative’ for 2 days; *plus* at least 3 features from ‘more active’, ‘more talkative’, ‘needed less sleep’ and ‘more creative/more ideas’; *plus* duration of a week or more; *plus***‘**needed treatment or caused problems at work’.
2. **BD, type II:** Ever ‘manic or hyper’ for 2 days OR ever ‘irritable/argumentative’ for 2 days; *plus* at least 3 features from ‘more active’, ‘more talkative’, ‘needed less sleep’ and ‘more creative/more ideas’; *plus* duration of a week or more.

Criteria for a classification of lifetime experience of probable MDD:

1. **Single probable episode of MDD:** Ever depressed/down for a whole week; *plus* at least two weeks duration; *plus* only one episode; *plus* ever seen a GP or a psychiatrist for ‘nerves, anxiety, depression’ OR ever anhedonic (unenthusiasm/uninterest) for a whole week; *plus* at least two weeks duration; *plus* only one episode; *plus* ever seen a GP or a psychiatrist for ‘nerves, anxiety, depression’.
2. **Probable recurrent MDD (moderate):** Ever depressed/down for a whole week; *plus* at least two weeks duration; *plus* at least two episodes; *plus* ever seen a GP (but not a psychiatrist) for ‘nerves, anxiety, depression’ OR ever anhedonic (unenthusiasm/uninterest) for a whole week; *plus* at least two weeks duration; *plus* at least two episodes; *plus* ever seen a GP (but not a psychiatrist) for ‘nerves, anxiety, depression’.
3. **Probable recurrent MDD (severe):** Ever depressed/down for a whole week; *plus* at least two weeks duration; *plus* at least two episodes; *plus* ever seen a psychiatrist for ‘nerves, anxiety, depression’ OR ever anhedonic (unenthusiasm/uninterest) for a whole week; *plus* at least two weeks duration; *plus* at least two episodes; *plus* ever seen a psychiatrist for ‘nerves, anxiety, depression’.

### Classification of chronic and multisite pain

At baseline assessment, participants were asked the question *“In the last month have you experienced any of the following that interfered with your usual activities?”* The response options related to seven specific sites of pain: headache, facial, neck or shoulder, back, stomach or abdominal, hip, and knee pain; and an eighth option of “pain all over the body”. Participants were permitted to select multiple options unless they reported having pain all over the body. Participants who indicated that they had pain were then asked whether the pain at each site had been present for more than 3 months (defined as chronic pain). Individuals were considered to have chronic multisite pain if they reported chronic pain in two body sites or more [3]. The chronic pain categories used for this study were: free of chronic pain; chronic single site pain; chronic pain at 2–3 sites; chronic pain at 4–7 sites; and chronic pain all over the body (widespread pain). Widespread pain was considered as a separate category because a participant who selected ‘pain all over the body’ may differ from one who selected seven individual sites of pain.

### Confounding factors

We considered sociodemographic factors, lifestyle factors and medical comorbidities as important potential confounders of the relationship between chronic pain syndromes and mood disorder. The sociodemographic factors included as covariates were: sex, age (as a continuous variable), ethnic group (defined by UK Biobank as white, black or black British, Asian or Asian British and other (including mixed, Chinese and other ethnic group)), employment status and area-based socioeconomic deprivation. Participants could select more than one option for their current employment status and for this study were categorised into mutually exclusive groups: paid employment/looking after home or family, retired (unless also selected not working due to sickness), unemployed/unpaid, not working due to sickness or disability, and student (if they selected no other category). Area-based socioeconomic status was assessed using the Townsend Index, a score derived from postcode of residence information on unemployment, car ownership, owner occupation and overcrowding [12]. A higher Townsend Index score reflects higher levels of deprivation. The Townsend score was categorised into quintiles based on frequency in this study population: quintile 1 represents the least deprived and quintile 5 the most deprived areas. Lifestyle factors considered as potential confounders included: body mass index (BMI) based on actual weight and height measurements taken at the assessment centre and categorised into: underweight <18.5 kg/m^2^, normal weight = 18.5-24.9 kg/m^2^, overweight =25.0-29.9 kg/m^2^, and obese ≥ 30.0 kg/m^2^ [13]; smoking status (current, former or never smokers); and frequency of alcohol consumption (daily/almost daily, 3–4 times per week, 1–2 times per week, 1–3 times per month, special occasions only and never). Doctor-diagnosis of cancer was self-reported using the touch screen questionnaire, and details of participants’ other medical conditions were based on self-report during a face to face interview with a trained nurse (UK Biobank field ID 20002). Based on previous work on multimorbidity in large population samples [14], a total of 43 long-term conditions were also classified (see Additional file 1: Table S1 for full details). For this study, painful conditions and psychiatric/depressive conditions were excluded, resulting in a total of 36 chronic comorbid conditions being considered. A count of medical comorbidities was constructed for each participant.

### Ethical approval

Participants provided full informed consent to participate in UK Biobank. This study was covered by the generic ethical approval for UK Biobank studies from the NHS National Research Ethics Service (approval letter dated 17^th^ June 2011, Ref 11/NW/0382).

### Statistical analysis

Initial descriptive analyses compared the extent of pain, demographic and lifestyle factors, and medical morbidity across the three mood disorder groups: BD, MDD and comparison (free of MDD and BD). Multinomial logistic regression models were constructed to quantify the relationship between the extent of chronic pain (independent variable) and mood disorder (dependent variable) relative to the comparison group. In order to examine the relative contribution of each group of putative confounding variables, four models were constructed: 1) no adjustment; 2) adjustment for demographic variables (age, sex, ethnicity, socioeconomic status); 3) as in model 2 plus lifestyle factors (BMI, smoking status and frequency of alcohol consumption); and 4) as in model 3 plus number of long-term conditions. In addition, possible interactions between the extent of pain and sex were formally tested in the unadjusted model (model 1). Results are presented as relative risk ratios (RRR) and 95% confidence intervals (95% CI). Binary logistic regression models were also constructed to quantify the relationship between the extent of chronic pain and BD relative to MDD; this analysis excluded participants in the comparison group. Four logistic regression models were constructed with identical levels of adjustment as those described above. The results are presented as odds ratios (OR) and 95% CI. All analyses were conducted using Stata V13.0 (StataCorp, College Station, Texas). Since the data were at least 99% complete for all variables used in this study imputation was not required; the number in each regression model varied according to the number of participants with complete data for the included variables.

## Results

### Study population

Of the 172,745 participants who were asked questions on lifetime history of depressive and manic features, 149,842 (86.7%) remained in the study and could be classified into one of the three mood disorder groups. Of these, 149,611 (99.8%) provided complete data on chronic pain status and constituted the study population. The study population was 53.3% female and the mean age was 56.7 years (standard deviation (SD) 8.2).

### Prevalence and characteristics of mood disorder groups

**Figure 1:**
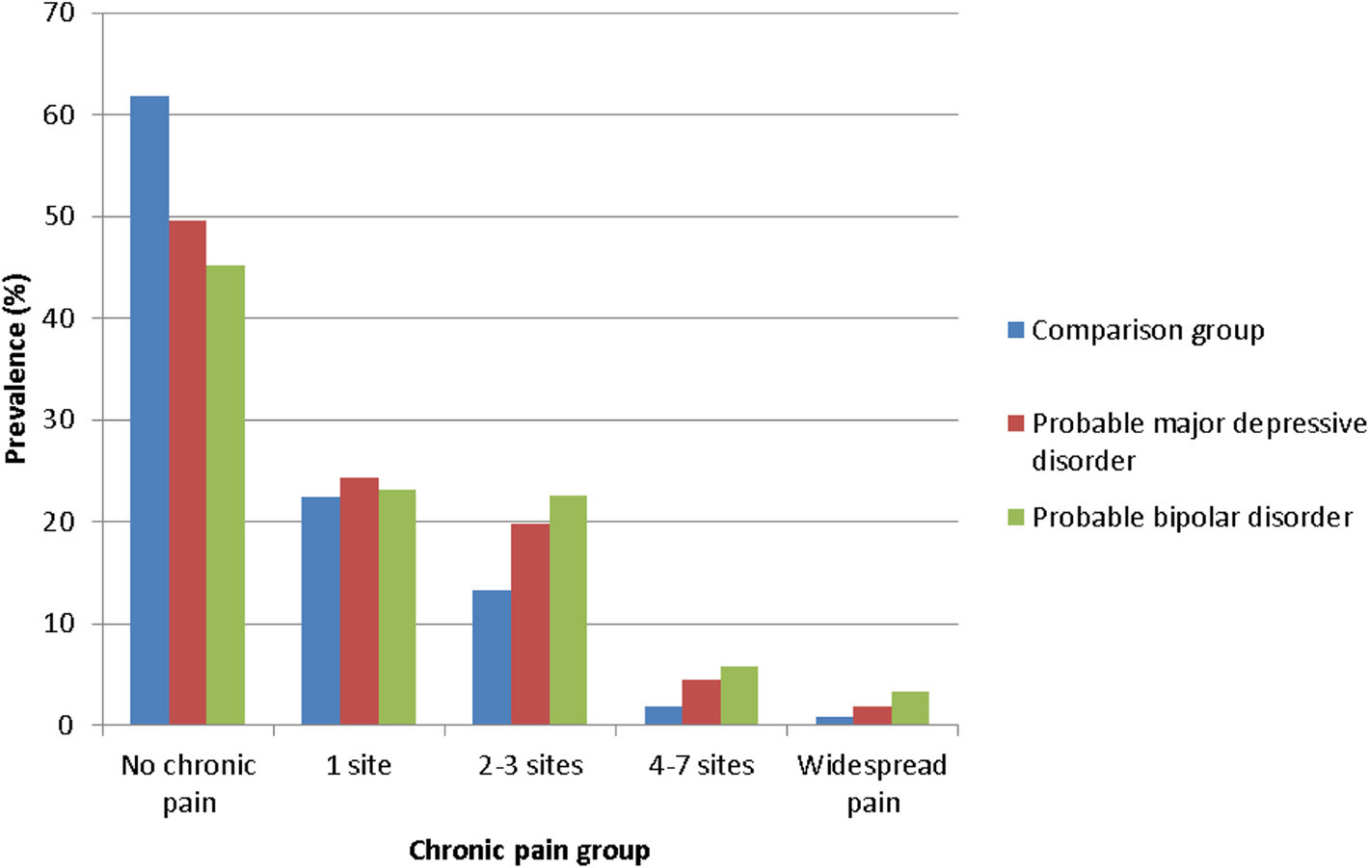
**Prevalence of chronic pain by mood group.** Bar chart depicting the proportion of participants in each of the multisite pain categories for each of the three mood disorder groups of interest.

Of the 149,611 participants included in the analysis, 31,814 (21.3%) were classified as MDD and 1,613 (1.1%) as BD and the remaining 116,184 (77.7%) comprised the non-mood disordered comparison group. The proportion of participants reporting single-site chronic pain was similar across the three groups (22.4%-24.3%; Table 1 and Figure 1). Although not a question of our analysis, it should be noted that all six regional sites of pain were most common in the BD group, second to the MDD group and then the comparison group. Similarly the prevalence of multisite pain (two or more sites or whole body) increased across the three groups from comparison group (15.8%) through MDD (26.1%) to BD (31.7%). Table 1 and Figure 1 provide further detail on the number and proportion of participants in each of the multisite pain categories across the groups; with the proportion reporting 2–3 sites, 4–7 sites and widespread pain clearly increasing from the comparison group, through MDD group to BD group.

**Table 1:** **Characteristics of participant according to mood disorder groups**

	Mood group*		
	**N = 149,611^**		
	**Comparison group**	**Probable depression**	**Probable bipolar disorder**
	**N = 116,184^**	**N = 31,814^**	**N = 1,613^**
	**N (%)**	**N (%)**	**N (%)**
**Chronic pain status**			
No chronic pain	71,800 (61.8)	15,766 (49.6)	729 (45.2)
1 site	26,061 (22.4)	7,736 (24.3)	373 (23.1)
2-3 sites	15,285 (13.2)	6,299 (19.8)	365 (22.6)
4-7 sites	2,068 (1.8)	1,416 (4.5)	93 (5.8)
Widespread pain	970 (0.8)	597 (1.9)	53 (3.3)
**Female**	58,395 (50.3)	20,490 (64.4)	790 (49.0)
**Townsend Index quintiles**			
1 (least deprived)	23,749 (20.5)	5,930 (18.7)	211 (13.1)
2	23,724 (20.5)	5, 921 (18.7)	213 (13.2)
3	23,324 (20.1)	6,285 (19.8)	267 (16.6)
4	23,004 (19.8)	6,507 (20.5)	364 (22.6)
5 (most deprived)	22,208 (19.1)	7,106 (22.4)	557 (34.6)
**Ethnicity**			
White	105,746 (91.4)	29,957 (94.5)	1,421 (88.8)
Asian/Asian British	3,955 (3.4)	584 (1.8)	66 (4.1)
Black/Black British	3,358 (2.9)	539 (1.7)	53 (3.3)
Other	2,683 (2.3)	623 (2.0)	61 (3.8)
**Employment status**			
In paid employment/look after home	66,418 (57.7)	18,111 (57.4)	880 (55.2)
Retired	42,985 (37.4)	10,403 (33.0)	401 (25.1)
Unemployed/unpaid	2,888 (2.5)	969 (3.1)	77 (4.8)
Not working due to sick	2,538 (2.2)	1,910 (6.1)	228 (14.3)
Student	269 (0.2)	140 (0.4)	9 (0.6)
**Smoking status**			
Never	66,360 (57.3)	15,988 (50.4)	686 (42.7)
Former	42,139 (36.4)	12,721 (40.1)	633 (39.4)
Current	7,288 (6.3)	3,036 (9.6)	286 (17.8)
**Frequency of alcohol intake**			
Daily/almost daily	24,246 (20.9)	6,281 (19.8)	326 (20.3)
3-4 times/week	27,110 (23.4)	6,720 (21.1)	267 (16.6)
1-2 times/week	29,736 (25.6)	7,653 (24.1)	352 (21.9)
1-3 times/month	12,624 (10.9)	4,053 (12.8)	201 (12.5)
Special occasions only	13,130 (11.3)	4,213 (13.3)	243 (15.1)
Never	9,279 (8.0)	2,878 (9.1)	220 (13.7)
**BMI categories**			
Underweight	558 (0.5)	161 (0.5)	15 (1.0)
Normal weight	38,218 (33.1)	9,950 (31.5)	460 (28.8)
Overweight	49,574 (43.0)	12,806 (40.5)	631 (39.5)
Obese	27,08 (23.4)	8,674 (27.5)	490 (30.7)
	**Mean (SD)**	**Mean (SD)**	**Mean (SD)**
**Age in years**	57.03 (8.2)	55.70 (8.0)	54.4 (8.1)
**Morbidity count**	0.88 (1.0)	1.09 (1.2)	1.15 (1.2)

^Overall number varies by the amount of missing data but 149,611 participants were eligible for analysis.

*Differences between groups were statistically significant at p < 0.001 for all variables (tests by Chi^2^ test for categorical variables and Kruskal Wallis test for continuous variables).

SD – standard deviation; BMI – body mass index.

The sociodemographic, lifestyle and morbidity characteristics of the groups are also reported in Table 1. Women comprised around half of the comparison and BD groups but almost two thirds of the MDD group. The mean age across the groups decreased from the comparison group through MDD to BD. Other notable differences included a greater proportion of the BD group being in the most deprived quintile of socioeconomic status compared to the MDD and comparison groups. Similarly, a greater proportion of the BD group compared to the MDD and comparison groups were not working due to sickness or disability, were current smokers, regarded themselves as “never” drinkers and were classified as obese. The mean number of medical morbidities showed a similar direction of relationship from BD through MDD to the comparison group (1.2, 1.1 and 0.9 respectively).

### Association between the extent of chronic multisite pain and mood disorder

The univariate multinomial regression model (model 1) confirmed that the likelihood of being classified as having a mood disorder (MDD or BD) increased with increasing number of sites affected by pain (Table 2). There was also a possible dose relationship whereby the likelihood of having BD (compared with MDD or no mood disorder) increased with number of pain sites.

**Table 2:** **Multinomial logistic regression models of the association between the extent of chronic multisite pain and mood disorder**

		RRR (95% CI)*	
**Model**		**Probable MDD**	**Probable BD**
1. No adjustments (n = 149,612)	No chronic pain	1	1
	1 site	1.35 (1.31, 1.39)	1.41 (1.24, 1.60)
	2-3 sites	1.88 (1.81, 1.94)	2.35 (2.07, 2.38)
	4-7 sites	3.12 (2.91, 3.34)	4.43 (3.55, 5.52)
	Widespread pain	2.80 (2.53, 3.11)	5.38 (4.04, 7.16)
2. Adjusted for sex, age, ethnicity, deprivation and employment status (n = 147,463)	No chronic pain	1	1
	1 site	1.32 (1.28, 1.37)	1.34 (1.18, 1.52)
	2-3 sites	1.74 (1.68, 1.80)	2.03 (1.78, 2.31)
	4-7 sites	2.54 (2.36, 2.73)	2.89 (2.29, 3.64)
	Widespread pain	2.23 (1.997, 2.49)	2.81 (2.07, 3.81)
3. Adjustments as for 2. plus BMI, smoking status and frequency of alcohol consumption (n = 145,945)	No chronic pain	1	1
	1 site	1.30 (1.26, 1.34)	1.29 (1.13, 1.47)
	2-3 sites	1.66 (1.61, 1.72)	1.92 (1.68, 2.19)
	4-7 sites	2.38 (2.21, 2.57)	2.62 (2.07, 3.33)
	Widespread pain	2.07 (1.85, 2.31)	2.61 (1.91, 3.55)
4. Adjustments as for 3. plus additional long term condition morbidity count (n = 145,518)	No chronic pain	1	1
	1 site	1.27 (1.23, 1.31)	1.27 (1.12, 1.45)
	2-3 sites	1.59 (1.54, 1.65)	1.84 (1.61, 2.10)
	4-7 sites	2.14 (1.99, 2.31)	2.39 (1.88, 3.04)
	Widespread pain	1.86 (1.66, 2.08)	2.37 (1.74, 3.24)

*Relative to non-mood disordered comparison group. Associations for individual variable level and the overall models were significant at p < 0.001.

RRR – relative risk ratio; 95% CI – 95% confidence interval; BMI – body mass index; MDD – major depressive disorder; BD – bipolar disorder.

**Figure 2:**
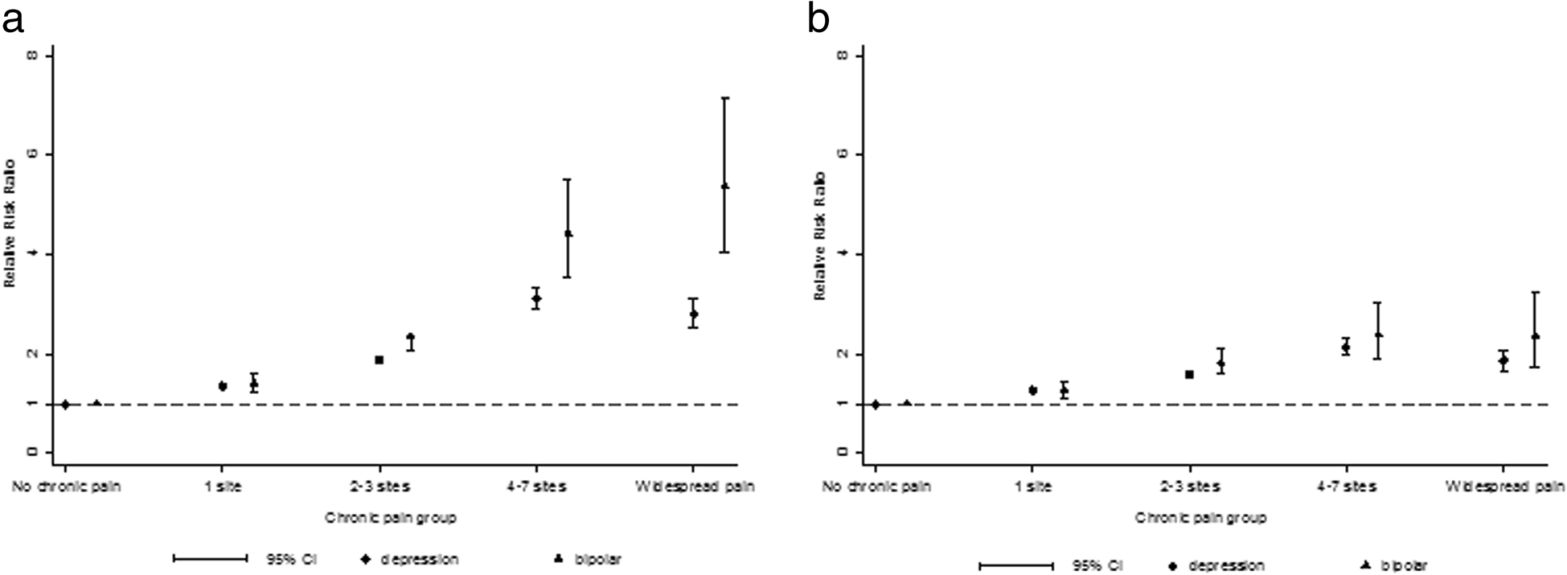
**Multinomial logistic regression model of the association between chronic pain and mood disorder.** Plot of the relative risk ratio and 95% confidence intervals of the association between extent of chronic pain and major depressive disorder and bipolar disorder for **a)** multinomial logistic regression model 1, unadjusted analysis, and **b)** multinomial logistic regression model 4, fully adjusted analysis for demographic variables (age, sex, ethnicity, socioeconomic status), lifestyle factors (BMI, smoking status and frequency of alcohol consumption), and number of long-term conditions.

The addition of potential sociodemographic confounders to the models attenuated these associations, especially for BD, but all of the associations remained statistically significant (p < 0.001). The addition of lifestyle covariates and comorbidity counts further attenuated the associations but there remained statistically significant associations between extent of chronic pain and both mood disorder groups (and particularly BD) with dose relationships from 0 to 4–7 pain sites (Table 2 and Figure 2).

There was no significant statistical interaction between sex and the extent of chronic multisite pain in relation to mood disorder groups, and therefore subsequent model stratification by sex was not carried out.

### Association between extent of chronic multisite pain and BD relative to MDD

Binary logistic regression models were constructed to quantify the association between the extent of chronic multisite pain and BD relative to MDD (rather than the comparison group) and are presented in Table 3. Model 1 (unadjusted analysis) found a significant dose–response relationship, with the odds of BD increasing with increasing extent of chronic pain. The odds of having BD increased to 1.92 for the group reporting widespread pain compared to no pain. Following adjustment for all confounding variables, this pattern of association remained; however, most of the relationships were no longer statistically significant and wide confidence intervals were observed. Reporting 2–3 sites of pain was the only category that remained significantly associated with having BD relative to MDD following full adjustment for all confounding variables (model 4: OR = 1.16). Again, there was no statistical interaction observed between chronic pain and sex in relation to mood group.

**Table 3:** **Logistic regression models of the association between the extent of chronic pain and probable bipolar disorder, relative to probable major depressive disorder**

**Model**		OR (95% CI*)	**P value^**	**P value^^**
1. No adjustments (n = 149,612)	No chronic pain	1		<0.001
	1 site	1.04 (0.92, 1.18)	0.520	
	2-3 sites	1.25 (1.10, 1.43)	0.001	
	4-7 sites	1.42 (1.13, 1.77)	0.002	
	Widespread pain	1.92 (1.44, 2.57)	<0.001	
2. Adjusted for sex, age, ethnicity, deprivation and employment status (n = 147,463)	No chronic pain	1		<0.001
	1 site	1.02 (0.90, 1.17)	0.724	
	2-3 sites	1.18 (1.03, 1.34)	0.018	
	4-7 sites	1.16 (0.92, 1.47)	0.210	
	Widespread pain	1.34 (0.99, 1.83)	0.061	
3. Adjustments as for 2. plus BMI, smoking status and frequency of alcohol consumption (n = 145,945)	No chronic pain	1		<0.001
	1 site	1.01 (0.88, 1.15)	0.898	
	2-3 sites	1.15 (1.01, 1.32)	0.039	
	4-7 sites	1.11 (0.88, 1.41)	0.372	
	Widespread pain	1.32 (0.97, 1.81)	0.079	
4. Adjustments as for 3. plus additional long term condition morbidity count (n = 145,518)	No chronic pain	1		<0.001
	1 site	1.01 (0.88, 1.15)	0.881	
	2-3 sites	1.16 (1.01, 1.33)	0.035	
	4-7 sites	1.12 (0.88, 1.43)	0.339	
	Widespread pain	1.34 (0.97, 1.83)	0.072	

*Relative to the probable MDD group.

^P-value for each level of chronic pain.

^^P-value for overall model.

OR – odds ratio; 95% CI – 95% confidence interval; BMI – body mass index.

## Discussion

Overall, we found that chronic pain was more commonly reported by individuals with MDD (50.4%) than for individuals without a history of mood disorder (38.2%), and was most common in those with BD (54.8%). Chronic pain affecting multiple parts of the body followed the same pattern. There was a positive relationship between the number of sites of chronic pain reported, up to 4–7 sites, and the risk of both BD and MDD. Widespread pain was associated with an increased risk of MDD but this was less than those who reported 4–7 sites of pain; the risk of BD was similar in groups reporting 4–7 sites of pain and widespread pain. These significant associations remained after adjusting for potential confounders: age, sex, ethnicity, socioeconomic status, employment status, BMI, smoking status, alcohol consumption and other long-term conditions.

Relative to the non-mood disordered group, at all levels of adjustment the RRR of MDD in individuals with 4–7 sites of pain was higher than it was in those with widespread pain; however, in the BD group the RRR is similar for the two pain groups. It is not clear why this might be. There are likely to be differences between individuals who select multiple sites of pain, which in essence must be widespread across the body, and those who select the single statement to agree that they have “pain all over the body”. These differences may be associated with psychological factors. Further work will be needed to try to understand the differences between these groups. In the logistic regression model the odds of having BD relative to MDD was only significantly higher in the group with 2–3 sites of pain (at each level of adjustment for confounders). We expected there to be an increased odds of BD relative to MDD as the extent of pain reported increased. However, the number of participants with BD and 4–7 sites of pain or widespread pain were 93 and 53, respectively, and when potential confounder variables were included in the adjusted models the power to detect an association would have decreased. When a suitable larger cohort is available, the association of multisite pain with BD in comparison to MDD should be studied further. This would help to elucidate reasons for the differences in the relationship of MDD and BD with multisite pain.

These findings are consistent with previous work on the association between chronic pain and MDD [1,2] but contribute to the field by reporting on multisite pain syndromes within a very large sample of adults aged 40–69 years. We were also able to compare associations across two mood disorder groups and a comparison group with no history of mood disorder within a single study and we adjusted for a wide range of potentially confounding factors. The association between chronic pain and BD has not been extensively studied compared to chronic pain and MDD. Our findings, alongside findings from recent clinical and epidemiological studies [7,8], suggest that chronic pain is more common in BD than in MDD, and that these relationships are independent of sociodemographic, lifestyle and comorbidity differences between BD and MDD groups. A meta-analysis of pooled studies of chronic pain prevalence in BD, resulting in a sample of over 170,000 individuals with BD, estimated the prevalence to be 23.7% [6], much lower than our finding in UK Biobank of chronic pain in one or more sites reported by 54.7% of the BD group and to that reported by Failde et al. (51.2%) [15]. This is likely to be due to the heterogeneity in both definitions of chronic pain and BD across the studies. Our analysis is the largest study to date to consider multisite self-reported pain in BD and we observed two sites or more of chronic pain in 31.7% of our BD group of 1,613 participants. Failde et al. (2013) found 44.5% of their BD study population reported pain at two sites or more [15]. The difference observed in prevalence of multisite pain may be explained by the study populations considered; in this study we have used a large general population based sample and applied BD classifications, whereas Failde et al. used a small clinical population diagnosed with BD and also experiencing depressive episodes. Patients with BD have been shown to be at a three-fold increased risk of clinically reported migraine compared to the general population [6]. Migraine or regional pain sites were not a focus of this study but interestingly we found that 13.5% of our BD group reported chronic “headache” compared to the 12.3% of the MDD and 5.2% of the control group. These initial findings warrant further investigation in the UK Biobank cohort.

Chronic pain adds to the already challenging treatment of MDD (and vice versa) [16,17]. Less is known on how chronic pain impacts the clinical management of BD and severe mental illness in general - chronic pain is also common in patients with schizophrenia [18]. Given the high prevalence of chronic pain, including multisite pain, in groups with MDD and BD it appears that there is certainly a role for assessing pain symptoms and considering pain management in the clinical decision making of these patients; both at secondary and primary care levels. A more effective role in managing patients with both physical and mental morbidities has been called for in both psychiatry [19,20] and general practice [21] and our findings support the need for this.

Mechanisms which might account for the association between chronic pain syndromes and depressive disorders are poorly understood, in part because of the heterogeneity within both clinical areas and the impact both conditions have on social, psychological and physical functioning. A complex interplay of biological, psychological and social factors within individuals contributes to the co-occurrence of mood disorders and chronic pain syndromes. Possible biological mechanisms include shared genetic predispositions to chronic pain and depression [22], common pathophysiological mechanisms (for example, in terms of abnormalities of serotonin, norepinephrine, and dopamine neurotransmission in the brain and within the peripheral nervous system) [23], as well as oxidative stress [24] and immunological theories, including a putative role for proinflammatory cytokines [25,26]. Additionally, the likelihood of an individual with chronic pain experiencing significant depressive features will be influenced by a broad range of psychological, personality and social support factors [27]. Given that we have identified that chronic multisite pain is more strongly associated with BD than MDD, and that this association persists after adjusting for several important confounders, including other physical morbidities, which are likely to impact on an individual’s chronic pain and mood symptom reporting, it is possible that there is a significant genetic and neurobiological overlap between severe mood disorders such as BD and vulnerability to chronic pain syndromes.

Although this study makes use of a very large population sample, some potential limitations are acknowledged. The data on chronic pain within UK Biobank was self-reported by participants, albeit using a structured question approach about seven specific sites of the body, which included upper and lower limb, and spinal pain as well as “pain all over the body” with a specified duration threshold of three months or longer, which is the widely accepted minimum duration for non-malignant pain to be classified as chronic [28]. We were limited to data collected by UK Biobank and a measure of pain severity was not available. The severity of individuals’ chronic pain symptoms is highly likely to impact on the outcomes of these patients. This will be investigated in future work by this research group. Our classifications of MDD and BD were based on self-report, rather than formal psychiatric examination, although, as with chronic pain assessment, a structured approach was used (see “Detailed information of the UK Biobank variables used for classifying lifetime experience of probable bipolar disorder and depression” subsection). Psychiatric diagnoses based on formal interviews were not possible within UK Biobank and our criteria for *probable* MDD and BD could be considered less stringent than formal diagnostic criteria. Nevertheless, as noted above, there were several important internal validators supporting our criteria for MDD and BD [11]. For example, the sex distributions of approximately 1:1 for probable BD and approximately 2:1 (women:men) for probable MDD are consistent with a large body of epidemiological work on lifetime rates of mood disorder in men and women [29,30]. Furthermore, the lifetime prevalence rates for bipolar disorder (1.1%) and recurrent major depressive disorder (21.3%) are consistent with other population-based lifetime estimates [11]. In the future, linkage of UK Biobank participants to routine health records will be possible and will enable these definitions to be studied further. This was a cross-sectional study and as such it was not possible to determine the temporal relationships between mood disorder and pain. However, future work planned will investigate these temporal relationships. There is also the potential for recruitment bias as individuals with mood disorder may be less likely to participate if they have pain, or vice versa.

## Conclusions

In summary, to our knowledge, this is the first assessment of the association between chronic multisite pain and both MDD and BD within a large and representative population cohort of middle to older aged adults. Chronic multisite pain commonly co-occurs with mood disorders, particularly BD, even after adjusting for multiple confounders. The risk of a probable mood disorder classification (both MDD and BD) also increased in proportion to the number of sites of chronic pain reported. These findings have implications for our understanding of the neurobiological relationship between chronic pain and mood disorders and, within a clinical context, are relevant because they highlight the importance of an adequate assessment of the nature and extent of pain in patients with BD or MDD. Further work on the UK Biobank cohort will assess whether there are shared genetic and biomarker risk factors for chronic pain and BD/MDD and, as prospective health record linkage data become available, we will assess the impact of pain and mood disorder comorbidity on outcomes for these highly morbid and often challenging clinical groups.

## Additional file

Additional file 1: Table S1. Long-term morbidity groupings.

## References

[1] Bair MJ, Robinson RL, Katon W, Kroenke K (2003). Depression and pain comorbidity: a literature review. Arch Intern Med.

[2] Arnow BA, Hunkeler EM, Blasey CM, Lee J, Constantino MJ, Fireman B, Kraemer HC, Dea R, Robinson R, Hayward C (2006). Comorbid depression, chronic pain, and disability in primary care. Psychosom Med.

[3] Carnes D, Parsons S, Ashby D, Breen A, Foster NE, Pincus T, Vogel S, Underwood M (2007). Chronic musculoskeletal pain rarely presents in a single body site: results from a UK population study. Rheumatology (Oxford).

[4] Kamaleri Y, Natvig B, Ihlebaek CM, Benth JS, Bruusgaard D (2008). Number of pain sites is associated with demographic, lifestyle, and health-related factors in the general population. Eur J Pain.

[5] Birgenheir DG, Ilgen MA, Bohnert AS, Abraham KM, Bowersox NW, Austin K, Kilbourne AM (2013). Pain conditions among veterans with schizophrenia or bipolar disorder. Gen Hosp Psychiatry.

[6] Stubbs B, Eggermont L, Mitchell AJ, De Hert M, Correll CU, Soundy A, Rosenbaum S, Vancampfort D: **The prevalence of pain in bipolar disorder: a systematic review and large-scale meta-analysis.** *Acta Psychiatr Scand* 2014. 10.1111/acps.12325 25098864

[7] Cerimele JM, Chan YF, Chwastiak LA, Unutzer J (2014). Pain in primary care patients with bipolar disorder. Gen Hosp Psychiatry.

[8] Subramaniam M, Abdin E, Vaingankar JA, Chong SA (2013). Prevalence, correlates, comorbidity and severity of bipolar disorder: results from the Singapore Mental Health Study. J Affect Disord.

[9] UK Biobank: **UK Biobank: Rationale, design and development of a large-scale prospective resource** [http://www.ukbiobank.ac.uk/resources/]

[10] Allen N, Sudlow C, Downey P, Peakman T, Danesh JEP, Gallacher J, Green J, Matthews P, Pell J, Sprosen T, Collins R, on behalf of UK Biobank (2012). UK Biobank: Current status and what it means for epidemiology. Health Policy and Technology.

[11] Smith DJ, Nicholl BI, Cullen B, Martin D, Ul-Haq Z, Evans J, Gill JM, Roberts B, Gallacher J, Mackay D, Hotopf M, Deary I, Craddock N, Pell JP (2013). Prevalence and characteristics of probable major depression and bipolar disorder within UK biobank: cross-sectional study of 172,751 participants. PLoS One.

[12] Townsend P (1987). Deprivation. J Soc Policy.

[13] Ul-Haq Z, Smith DJ, Nicholl BI, Cullen B, Martin D, Gill JM, Evans J, Roberts B, Deary IJ, Gallacher J, Hotopf M, Craddock N, Mackay DF, Pell JP (2014). Gender differences in the association between adiposity and probable major depression: a cross-sectional study of 140,564 UK Biobank participants. BMC Psychiatry.

[14] Barnett K, Mercer SW, Norbury M, Watt G, Wyke S, Guthrie B (2012). Epidemiology of multimorbidity and implications for health care, research, and medical education: a cross-sectional study. Lancet.

[15] Failde I, Duenas M, Aguera-Ortiz L, Cervilla JA, Gonzalez-Pinto A, Mico JA (2013). Factors associated with chronic pain in patients with bipolar depression: a cross-sectional study. BMC Psychiatry.

[16] Lin EH, Katon W, Von Korff M, Tang L, Williams JW, Kroenke K, Hunkeler E, Harpole L, Hegel M, Arean P, Hoffing M, Della Penna R, Langston C, Unutzer J, IMPACT Investigators (2003). Effect of improving depression care on pain and functional outcomes among older adults with arthritis: a randomized controlled trial. JAMA.

[17] Kroenke K, Shen J, Oxman TE, Williams JW, Dietrich AJ (2008). Impact of pain on the outcomes of depression treatment: results from the RESPECT trial. Pain.

[18] Smith DJ, Langan J, McLean G, Guthrie B, Mercer SW: **Schizophrenia is associated with excess multiple physical-health comorbidities but low levels of recorded cardiovascular disease in primary care: cross-sectional study.** *BMJ Open* 2013, **3**(4). doi:10.1136/bmjopen-2013-002808. Print 2013. 10.1136/bmjopen-2013-002808 PMC3641427 23599376

[19] Elman I, Zubieta JK, Borsook D (2011). The missing p in psychiatric training: why it is important to teach pain to psychiatrists. Arch Gen Psychiatry.

[20] Langan J, Mercer SW, Smith DJ (2013). Multimorbidity and mental health: can psychiatry rise to the challenge?. Br J Psychiatry.

[21] Mercer SW, Gunn J, Bower P, Wyke S, Guthrie B (2012). Managing patients with mental and physical multimorbidity. BMJ.

[22] Mahatme SS, Dhavale HS, Patkar AP (1989). Study of correlation of intensity of symptoms with stressful life events in depressed patients. Indian J Psychiatry.

[23] Goldenberg DL (2010). Pain/Depression dyad: a key to a better understanding and treatment of functional somatic syndromes. Am J Med.

[24] Arora V, Kuhad A, Tiwari V, Chopra K (2011). Curcumin ameliorates reserpine-induced pain-depression dyad: behavioural, biochemical, neurochemical and molecular evidences. Psychoneuroendocrinology.

[25] Illi J, Miaskowski C, Cooper B, Levine JD, Dunn L, West C, Dodd M, Dhruva A, Paul SM, Baggott C, Cataldo J, Langford D, Schmidt B, Aouizerat BE (2012). Association between pro- and anti-inflammatory cytokine genes and a symptom cluster of pain, fatigue, sleep disturbance, and depression. Cytokine.

[26] Dowlati Y, Herrmann N, Swardfager W, Liu H, Sham L, Reim EK, Lanctot KL (2010). A meta-analysis of cytokines in major depression. Biol Psychiatry.

[27] Hall AM, Kamper SJ, Maher CG, Latimer J, Ferreira ML, Nicholas MK (2011). Symptoms of depression and stress mediate the effect of pain on disability. Pain.

[28] Merskey H, Bogduk N, IASP Task Force (1994). Taxonomy. Classification of Chronic Pain: Second ed.

[29] Kessler RC, McGonagle KA, Swartz M, Blazer DG, Nelson CB (1993). Sex and depression in the National Comorbidity Survey. I: Lifetime prevalence, chronicity and recurrence. J Affect Disord.

[30] Weissman MM, Bland RC, Canino GJ, Faravelli C, Greenwald S, Hwu HG, Joyce PR, Karam EG, Lee CK, Lellouch J, Lepine JP, Newman SC, Rubio-Stipec M, Wells JE, Wickramaratne PJ, Wittchen H, Yeh EK (1996). Cross-national epidemiology of major depression and bipolar disorder. JAMA.

