# Supplementary material for: Chronic multisite pain in major depression and bipolar disorder: cross-sectional study of 149,611 participants in UK Biobank
Source: BMC Psychiatry. 2014 Dec 10;14:350. doi: 10.1186/s12888-014-0350-4 (PMC4297369; doi:10.1186/s12888-014-0350-4)
Supplement: Additional file 1: Table S1. — Long-term morbidity groupings. [file 12888_2014_350_MOESM1_ESM.docx]

**Additional file 1: Table S1 – Long-term morbidity groupings**

| **Morbidity grouping^** | **Conditions included** |
| --- | --- |
| 1. **Painful conditions*** | Back pain |
|  | Joint pain |
|  | Headaches (not migraine) |
|  | Sciatica |
|  | Plantar fasciitis |
|  | Carpal tunnel syndrome |
|  | Fibromyalgia |
|  | Arthritis |
|  | Shingles |
|  | Disc problem |
|  | Prolapsed disc/slipped disc |
|  | Spine arthritis/spondylitis |
|  | Ankylosing spondylitis |
|  | Back problem |
|  | Osteoarthritis |
|  | Gout |
|  | Cervical spondylosis |
|  | Trigeminal neuralgia |
|  | Disc degeneration |
|  | Trapped nerve/compressed nerve |
| 1. **Hypertension** | Hypertension |
|  | Essential hypertension |
| 1. **Depression*** | Depression |
|  | Postnatal depression |
| 1. **Asthma** | Asthma |
| 1. **Coronary Heart Disease** | Heart attack/MI |
|  | Angina |
| 1. **Treated dyspepsia** | Gastro-oesophageal reflux (GORD)/gastric reflux |
|  | Oesophagitis /Barrett's oesophagus |
|  | Gastric stomach ulcers |
|  | Gastric erosions/gastritis |
|  | Duodenal ulcer |
|  | Dyspepsia/indigestion |
|  | Hiatus hernia |
|  | Helicobacter pylori |
| 1. **Diabetes** | Diabetic nephropathy |
|  | Diabetic neuropathy/ulcers |
|  | Diabetes |
|  | Type 1 diabetes |
|  | Type 2 diabetes |
|  | Diabetic eye disease |
| 1. **Thyroid disorders** | Thyroid problem (not cancer) |
|  | Hyperthyroidism/thyrotoxicosis |
|  | Hypothyroidism/myxoedema |
|  | Graves’ disease |
|  | Thyroid goitre |
|  | Thyroiditis |
| 1. **Rheumatoid arthritis, other inflammatory polyarthropathies, systemic connective tissue disorders and systemic autoimmune disorders** | Myositis/myopathy |
|  | Systemic Lupus Erythematosus |
|  | Connective tissue disorder |
|  | Sjogren’s syndrome/sicca syndrome |
|  | Dermatopolymyositis |
|  | Scleroderma/systemic sclerosis |
|  | Rheumatoid arthritis |
|  | Psoriatic arthropathy |
|  | Dermatomyositis |
|  | Polymyositis |
|  | Polymyalgia Rheumatica |
|  | Malabsorption/coeliac disease |
| 1. **Chronic Obstructive Pulmonary Disease (COPD)** | COPD/chronic obstructive airways disease |
|  | Emphysema/chronic bronchitis |
|  | Emphysema |
| 1. **Anxiety, other neurotic, stress related and somatoform disorders*** | Anxiety/panic attacks |
|  | Nervous breakdown |
|  | Post-traumatic stress disorder |
|  | Obsessive compulsive disorder |
|  | Stress |
|  | Insomnia |
|  | Psychological/psychiatric problem |
| 1. **Irritable bowel syndrome** | Irritable bowel syndrome |
| 1. **Alcohol problems*** | Alcohol dependency |
|  | Alcoholic liver disease/alcoholic cirrhosis |
| 1. **Other psychoactive substance abuse*** | Opioid dependency |
|  | Other substance abuse/dependency |
| 1. **Treated constipation** | Constipation |
| 1. **Stroke and Transient Ischaemic Attack (TIA)** | Stroke |
|  | TIA |
|  | Subarachnoid haemorrhage |
|  | Brain haemorrhage |
|  | Ischaemic stroke |
| 1. **Chronic kidney disease** | Polycystic kidney |
|  | Diabetic nephropathy |
|  | Renal/kidney failure |
|  | Renal failure requiring dialysis |
|  | Renal failure not requiring dialysis |
|  | Kidney nephropathy |
|  | Immunoglobulin A (IgA) nephropathy |
| 1. **Diverticular disease of intestine** | Diverticular disease/diverticulitis |
| 1. **Atrial fibrillation** | Atrial fibrillation |
| 1. **Peripheral vascular disease** | Peripheral vascular disease |
|  | Leg claudication/intermittent claudication |
| 1. **Heart failure** | Cardiomyopathy |
|  | Hypertrophic cardiomyopathy |
|  | Heart failure/pulmonary oedema |
| 1. **Prostate disorders** | Prostate problem (not cancer) |
|  | Enlarged prostate |
|  | Benign prostatic hypertrophy |
| 1. **Glaucoma** | Glaucoma |
| 1. **Epilepsy** | Epilepsy |
| 1. **Dementia** | Dementia/Alzheimer/cognitive impairment |
| 1. **Schizophrenia (and related non-organic psychosis) and bipolar disorder*** | Schizophrenia |
|  | Mania/bipolar disorder/manic depression |
| 1. **Psoriasis or eczema** | Eczema/dermatitis |
|  | Psoriasis |
| 1. **Inflammatory bowel disease** | Inflammatory bowel disease |
|  | Crohn’s disease |
|  | Ulcerative colitis |
| 1. **Migraine** | Migraine |
| 1. **Chronic sinusitis** | Chronic sinusitis |
| 1. **Anorexia or bulimia*** | Anorexia, bulimia/other eating disorder |
| 1. **Bronchiectasis** | Bronchiectasis |
| 1. **Parkinson's disease** | Parkinson's disease |
| 1. **Multiple sclerosis** | Multiple sclerosis |
| 1. **Viral Hepatitis** | Infective/viral hepatitis |
|  | Hepatitis B |
|  | Hepatitis C |
|  | Hepatitis D |
|  | Hepatitis E |
| 1. **Chronic liver disease** | Oesophageal varices |
|  | Non infective hepatitis |
|  | Liver failure/cirrhosis |
|  | Primary biliary cirrhosis |
| 1. **Osteoporosis~** | Osteoporosis |
| 1. **Chronic fatigue syndrome~** | Chronic fatigue syndrome |
| 1. **endometriosis~** | Endometriosis |
| 1. **Meniere disease~** | Meniere disease |
| 1. **Pernicious Anaemia~** | Pernicious anaemia |
| 1. **Polycystic ovaries~** | Polycystic ovaries |
| 1. **Cancer** | Lifetime diagnosis |
| ^ Self-report lifetime diagnosis by doctor recorded by nurse-led interview (UK Biobank data field 20002), except cancer diagnosis which was reported by touch-screen questionnaire (UK Biobank data field 2453). The list of disease groupings was based on Barnett K, Mercer SW, Norbury M, Watt G, Wyke S, Guthrie B: Epidemiology of multimorbidity and implications for health care, research, and medical education: a cross-sectional study. Lancet 2012, 380(9836):37–43.  ~Plus other conditions considered as long-term, requiring medication and that had a prevalence of ≥0. 1% in the whole UK Biobank cohort.  * Painful and psychiatric conditions were not included in the morbidity count for this study; resulted in a total of 36 morbidities included. | |
